# Drug Safety and Suicidality Risk of Chronic Pain Medications

**DOI:** 10.3390/ph16101497

**Published:** 2023-10-20

**Authors:** Osman Syed, Predrag Jancic, Adam B. Fink, Nebojsa Nick Knezevic

**Affiliations:** 1 Advocate Illinois Masonic Medical Center, Department of Anesthesiology, Chicago, IL 60657, USA; osman.syed@midwestern.edu (O.S.); prjancic@gmail.com (P.J.); adamfinkmd@gmail.com (A.B.F.); 2 Chicago College of Osteopathic Medicine, Midwestern University, Downers Grove, IL 60515, USA; 3 Harborview Medical Center, Department of Anesthesiology and Pain Medicine, University of Washington, Seattle, WA 98104, USA; 4 Department of Anesthesiology, University of Illinois, Chicago, IL 60612, USA; 5 Department of Surgery, University of Illinois, Chicago, IL 60612, USA

**Keywords:** drug safety, chronic pain, suicidality, opioids, benzodiazepines, antidepressants, gabapentinoids, prednisone

## Abstract

Chronic pain is one of the main leading causes of disability in the world at present. A variety in the symptomatology, intensity and duration of this phenomenon has led to an ever-increasing demand of pharmacological treatment and relief. This demand for medication, ranging from well-known groups, such as antidepressants and benzodiazepines, to more novel drugs, was followed by a rise in safety concerns of such treatment options. The validity, frequency, and diversity of such concerns are discussed in this paper, as well as their possible effect on future prescription practices. A specific caution is provided towards the psychological safety and toll of these medications, regarding suicidality and suicidal ideation. Most significantly, this paper highlights the importance of pharmacovigilance and underscores the necessity of surveillance programs when considering chronic pain medication.

## 1. Introduction

Current research estimates that 1.9 billion [1] individuals, and perhaps up to 30% [2] of the world’s population, suffers from chronic pain, which is a leading cause of disability globally [3]. Although defined as pain that persists or recurs for longer than 3 months, the taxonomy [4,5] of chronic pain conditions has evolved as the clinical and scientific understanding of the syndromes and diseases that comprise the condition have grown. At present, chronic pain is understood to be a form of chronic disease, underpinned by nociceptive, neuropathic, and nociplastic mechanisms that can overlap, and/or exist on a continuum [2].

The clinical symptomatology and syndromes [4] of chronic pain are as heterogenous as the pathophysiological process underpinning them and as varied as the pharmacological attempts to alleviate them. NSAIDs, opioids, benzodiazepines, gabapentinoids, multiple generations of antidepressants, steroids, and antipsychotic medications are all utilized as treatments [6]. Many of these medications, irrespective of the indication they are being used for, have well documented associations with self-harm, suicidality, and overdose [7,8,9,10]. This is further complicated by the association and comorbidity that exists between chronic pain and psychiatric conditions, such as depression [11], bipolar disorder [12], and schizophrenia [13], which are associated with self-harm, suicidality [14,15], and overdose [16] themselves. Additionally, these patients may only experience relief from their symptoms when using these medications, some of which have a well demonstrated addictive potential [17,18]. The totality of these factors renders this population vulnerable to both intentional and unintentional misuse if their medications naturally pose drug safety concerns.

Given this reality, therapeutic drug monitoring and pharmacovigilance are of the utmost importance in this patient population. In 2018, an interdisciplinary group of clinicians developed consensus-based recommendations regarding urine drug monitoring for chronic pain patients who are treated with opioids [19]. While the combination of urinary drug testing and immunoassays has been found to be appropriate in some studies [20], others concluded that immunoassays designed to detect opioids lack sensitivity and produce false negative results in up to 21% of cases [21]. These difficulties have been described in the case of therapeutic drug monitoring of benzodiazepines [22] as well. The current lack of effective, large-scale programs combined with the disruption in point of care monitoring during the pandemic has hindered progress in the realm of drug safety for chronic pain patients.

The aim of this article is to provide an updated epidemiological review and expand on the key findings as they relate to drug safety concerns associated with the most common medications used to treat chronic pain. Additionally, this article reviews current clinical trials and provides an insight into novel treatments.

## 2. Epidemiology

Chronic pain is a debilitating condition affecting around 20% of the US population [23,24], exhibiting nearly the same prevalence within the European population [25]. Broken down more individually, chronic pain affects 13–50% of adults in the UK population, of which 10.4–14.3% live with moderate to severe disability [1]. That number rises from 27% to 30% in countries such as Italy and Norway, respectively [25]. This type of variation was also seen in parameters such as pain intensity and the causes of said pain [25].

High-impact chronic pain, defined not by pain intensity but by its negative impact on daily life, affects 8% of the US population over the age of 50 [23,26], a number further corroborated by previous population studies [27]. High-impact chronic pain is most often attributed to diseases that are age-related, such as diabetes and neuropathy, osteoarthritis, and post-stroke pain [28].

The current literature suggests that a bidirectional relationship between chronic pain and mental disorders, including suicidality, exists [29]. An estimated 20% of people with chronic pain show some form of suicidal ideation, with the risk of death by suicide within this group being more than two times higher compared to the general population [30]. More precisely, parameters such as death wishes, suicide ideation, and plans, attempts and deaths by suicide were all significantly more prevalent in people with pain than those without (24.9%, 23.0%, 9.0%, 15%, and 2%, respectively) [31].

## 3. Socio-Demographic Characteristics

The socio-demographic parameters associated with a higher incidence of chronic pain are well known and established, with the main groups being age, sex, education, and marital status as well as socio-economic standing [1,32].

Age has consistently been a proven important risk factor for the development of chronic pain. Different studies suggest a wide range of prevalence of chronic pain amongst the elderly [23,33]. However, all are in agreement that the prevalence in the elderly is significantly higher compared to the younger population [34]. In addition, age has been reported as a specific risk factor for high-impact chronic pain [35].

Men are less likely to report the experience of chronic pain than women [36]. Similarly, women are more likely to seek diagnosis and treatment compared to men, which could in turn cause possible misleading statistics [37]. Women have a tendency to maladaptive coping strategies to pain, predisposing them to a possibility of developing chronic pain [38].

In women, schooling of 12 or more years was associated with a lower prevalence of chronic pain. In the same light, unemployed women show a higher prevalence of chronic pain compared to their employed counterparts. Marital status showed influence on both sexes, where an increased prevalence of chronic pain was noted in divorced or widowed women; yet, conversely, it decreased in single men compared to married, divorced, or widowed men [39].

Chronic pain is a well-established follower of lower socio-economic status and a poorer quality of life [40]. Those who are socio-economically deprived are not only more likely to experience chronic pain but also more likely to experience more severe pain and a greater level of pain-related disability [26]. It has also been shown that average pain intensity and pain-related disability was greater among African American than White respondents, and it decreased as wealth increased [26]. The prevalence of chronic pain in the homeless substantially exceeds those reported in the general population, at 59.3%, with the mean duration of that pain sitting at slightly over 6 years [41].

When it comes to suicidality and chronic pain, a review of the currently available literature reveals a myriad of research suggesting that suicidal ideation and suicidal attempts are more common in patients with chronic pain [30]. In addition to that, although assumed logical, patients with painful conditions are at a greater risk of suicide compared to patients with non-painful conditions [42].

Well-known risk factors for suicide in the general population include family history of suicide, previous attempts, comorbid depression, and female sex [43,44]; however, less is known for pain-specific risk factors.

A comprehensive study published in 2006 suggested that factors such as location, type, duration, and intensity of pain all play a role in increasing the risk of suicide in patients with chronic pain [30]. Headaches, especially migraines, and non-arthritic chronic pain were related to an increase in suicidal ideation and suicidal attempts, respectively [45]. To further point out the importance of the type of pain, a 2013 study showed that migraines, psychogenic pain, and back pain were associated with a significantly higher likelihood of suicide completion, while neuropathic pain and other types of headaches were not [46].

However, it is of importance to note that there is a vast amount of literature that suggest that sex [47,48], marital status [47,49], level of education [50], and age [47,51] are not definitive risk factors for suicide, suggesting a multifactorial etiology to suicidality.

Patients with simultaneous chronic pain and substance use disorders (SUDs) are a more complex topic. Physicians must focus on both chronic pain treatment as well as issues of medication safety and misuse. The data suggest that the current prevalence of SUDs amongst patients with chronic pain ranges from 3% to 48%, and that the lifetime SUD rate is somewhere between 16% and 74% [52]. Additionally, up to 11.5% of chronic pain patients that take opioids develop aberrant medication-related behaviors [53]. Other studies suggest that patients with chronic pain have a 2 to 3 times more likely chance of developing SUDs, compared to individuals without pain [54].

## 4. Pharmacological Treatment and Suicidality

### 4.1. Antidepressants

Overall, the use of antidepressants has increased in recent decades, with SSRIs leading this trend change since their discovery [55,56].

Though not the main indication, one of the treatment options for chronic pain have historically been antidepressants, with TCAs, SSRIs, and SNRIs being in the forefront of therapeutic options [57,58]. Patients with chronic pain are often treated for a prolonged period of time, usually with more than one type of medication [59], all of which have a vast side effect profile [60]. These factors, in combination with the psychological strain that these patients are exposed to [61], can lead to a mental state prone to suicidal thinking and ideation [51,62].

A variety of information currently exists on the topic of suicidality rates in correlation to antidepressant use, suggesting negative, positive and no correlation at all [63,64,65,66]. Randomized controlled trials (RCTs), baring the highest level of evidence, showed contradictory results, ranging from a significantly increased suicide risk while using antidepressants [67,68,69,70] to seemingly no change in such risk [71,72,73]. Nonetheless, the vast amount of literature showing that a relationship between these parameters might exist is worth mentioning.

A meta-analysis published in 2006 showed that the use of antidepressants in pediatric patients presented a moderate increase in suicide risk [74]. Some of the explanations proposed for this include misdiagnosing bipolar states for unipolar depression, with a possible induction of mania [75], age-dependent antidepressant-induced mania [76,77], or possible neuroanatomical and biochemical differences between the adolescent and the adult brain [78,79,80].

Additionally, a 2009 study showed a highly age-dependent relationship between the use of antidepressants and suicidality. The research showed that young adults (aged 25 and younger) showed a higher suicidality rate on these medications, in adherence with findings in children and adolescents. Conversely, the use of antidepressants in adults (aged 25 and older) showed a protective factor against suicidality [73]. This discrepancy in response to the initiation of antidepressant treatment was later reproduced many times in the literature, further supporting the caution of prescribing these medication to children and younger adults [72,81]. However, it is of high importance to mention that, with an increased risk of suicide in untreated depression and no concrete proof of association between pharmacotherapy and changes suicidality, physicians are still urged to prescribe SSRIs to affected minors [63].

The favored adverse effect profile of SSRIs seems to be translating into the scopes of suicidality as well, according to a 2020 meta-analysis that showed no definitive association between the use of these medications and a heightened risk of suicide [82]. However, the same publication showed that such a correlation exists with the use of new-generation antidepressants (venlafaxine, bupropion, and mirtazapine), findings congruent with previously published studies [70,83,84]. Moreover, SSRIs have been shown to be relatively safe in possible overdose cases, with mirtazapine being slightly more toxic than its counterparts. However, compared to other SSRIs and different groups of antidepressants such as TCAs, mirtazapine has been shown to have a very favorable cardiovascular adverse event profile [85,86]. The use of mirtazapine as an off-label medication for the treatment of insomnia has put this drug in correlation with decreased suicidality trends, more so by decreasing insomnia as a known suicidality risk, rather than having direct effect on suicidality and suicidal ideation [87].

The time of onset of suicidality in relation to depression treatment is of key importance to understanding the possible connection between these states. Suicidal ideation and behavior can initially develop during depression treatment, expressed by the term treatment-emergent suicidal ideation (TESI). In the case of the already present suicidality that changes in intensity in reaction to treatment, the term treatment worsening of suicidal ideation (TWOSI) fits best [88,89]. Even though these terms have been linked to a poorer socio-economic status and genetic predisposition [89,90,91], the literature suggests that more research is needed to conclude such a relationship. Some nation-based studies have reproduced this correlation between the use of antidepressants and the onset of suicidal attempts [92,93]. Other nation-based studies relating to suicide rates and suicidality in association with antidepressant use can be found in Table 1.

### 4.2. Opioids

Among all the medication classes discussed in this paper, opioids by far have received the most attention from the public in recent years. The socio-economic factors that are related to opioid use may directly contribute to the inherent risk of suicide with opioids. It has been theorized that the diminishment in the middle class and the general economic stress of workers has led to a use of opioids as a coping mechanism [97]. Furthermore, patients who are on opioid medications for extended periods of time have significantly increased risks of developing depression [98,99]. Both the relationship of opioid use with hardship, as well as the relationship between use and developing depression, seem likely candidates as the causes of opioid-related suicides.

The national center on health statics reported 80,311 overdose deaths involving opioids in 2021, about 75.4% of all lethal overdoses [100]. In October 2017, the opioid crisis was declared a public health emergency by the US government. The CDC reported that, in 2020, about 143 million prescriptions were written in the United States. This follows a general trend of declining prescriptions in the USA since 2012 [101]. While the general trend of declining opioid prescriptions may mean that certain communities are less encumbered by the societal burden of treating addiction, this effect is very clearly disproportionately applied across the USA. The CDC makes note of county-level trends that are extremely different than the nationwide averages, with certain counties reaching prescription rates (per 100 persons) up to nine times higher than the average (43.3 prescriptions per 100 persons) [101].

Prescription maps of the USA illustrate these trends, showing the dramatic patchwork of opioid prescription rates across the country. There are multiple theories that attempt to specifically define the risk factors that these areas share. Theories include income, race, and density of people (rural vs. urban development).

In a 2018 brief by the US department of Health and Human Services, individuals below the poverty line were over twice as likely to have an opioid use disorder compared to someone 200% above of the poverty line [102]. Regarding a population-level study, a 2017 study on the macroeconomic effects on local opioid abuse found that opioid deaths and emergency department visits rose when unemployment increased within a specific county. Specifically, their data showed that a single percentage point in county-level unemployment increases opioid fatalities by 3.55%, while a one standard deviation change in unemployment corresponds to a 9.2% increase in fatal overdoses [103]. This also correlates with similar trends seen in mental health during periods of general economic downturn. For example, the general use of prescription mental health medications was seen to have increased across all workers following the 2008 recession, and, at a local level, factories with more layoffs experienced greater increases in demand for psychiatric medication as well [104]. While we cannot draw a conclusion that increases in demand for mental health services during periods of economic downturn is related specifically to an increase in suicidal opioid overdoses, the general risk to one’s mental health is shown to be increased both in the realm of opioid misuse as well as crises requiring psychiatric intervention.

Regarding the effects of community structures and opioid misuse, a 2020 study investigated the relationship of income inequality and opioid prescription rates. The authors specifically investigated the variables of residential stability, defined as the level of connectedness between residents as assessed through people staying housed in the same place, as well as the variable of social isolation among individuals [105]. The authors were able to quantify these variables in a couple different ways. The Gini index was used as a measure of income inequality, residential stability was defined by the percentage of housing occupied by owners, and social isolation was indexed via the percentage of individuals who are disabled, the percentage of individuals who live alone, and those living in poverty. These variables were chosen as the demographic studied was those enrolled in Medicare, a program only available predominately to those above 65 years old, or with end-stage renal disease, amyotrophic lateral sclerosis, and those with severe disabilities. The authors found that high levels of income inequality are associated with low levels of residential stability and high levels of social isolation. Both variables in turn are related to more opioid prescriptions [105]. An important note, however, is that income inequality was not directly related to prescription rates, but rather alters residential stability and social isolation, which then in turn drive prescriptions. Furthermore, these conclusions dramatically change when the urban vs. rural dichotomy is considered. The study found that social isolation has a much larger mediating effect in urban areas, and residential stability plays a larger effect in rural areas [105]. Once again, although a definitive conclusion cannot be drawn between income inequality and opioid suicidality, the relationship between inequality and opioid use, and the relationship between opioid use and suicidality likely correlate.

These conclusions across the last few paragraphs can be further related to the fact that income inequality has a correlation to depression and suicidality [106]. As these papers have shown, these inequities lead to more opioids being used, and there are likely going to be more completed suicides due to the relationship between access to a highly lethal suicide means and completion [107].

Looking beyond economic factors, sex differences are also quite profound in opioid-related suicide. In general, women attempt suicide at greater rates then men but die by suicide at significantly lower rates. In the context of opioid-related suicide, a large cross-sectional study found that women comprise 54% of lethal suicides with opioids [108]. This may be further contextualized by a higher incidence in concomitant mental health complaints among women being treated for opioid dependence [109], as well as women with opioid use disorder reporting usage to cope with negative emotions at a higher rate [110]. Of note, the overall intentional overdose rate for all medications decreased between 2012 and 2019, but increased among African American women [111]. This may be a reflection on the general change in demographics that comprises the opioid crisis in the USA.

A 2022 cross-sectional study looking at fatal drug overdose rates found that the overdose death rate among the African American population increased to 36.8 per 100,000 people, which corresponds to the first time since 1999 that the African American overdose mortality was above that of the White population. The study further showed that American Indian and Alaskan natives had the highest rates per capita [112].

If the above-mentioned is to be believed, then a rapid increase in opioid usage, as indicated by the dramatic changes in lethality among different demographics, should correlate with respective changes in the intentional suicide rate in the respective groups. A 2010 cross-sectional study examining multiple-cause-of-death data from the National Center for Health Statistics found a disparity in the data quality for suicides in the African American population, as well as in the Hispanic population to a lesser degree. The study estimated an over two times likelihood in suicide misclassification as compared to that of the White population. It further stated that decedents without a psychiatric diagnosis on their death certificates have a three times higher chance of misclassification [113].

It has been noted that there is a multifactorial incidence of lower access to psychiatry in minority populations [114,115,116], along with lower rates of naloxone access in minority populations. Therefore, it is possible that, among minority patients who intentionally overdose and are not revived, more suicides will be misclassified as unintentional deaths. In a population study of New York City opioid users, researchers found significant differences in naloxone use and training. The data showed that African American participants had a 0.4 odds ratio of naloxone training, African American people above 50 years of age had an odds ratio of 0.2, and African American women scored 0.27 compared to White women [117]. A similar study of people who inject drugs in Los Angeles and San Francisco found that Latin (aRR = 0.53; 95% CI: 0.39, 0.72) and African American (aRR = 0.73; 95% CI: 0.57, 0.94) vs. White study participants were negatively associated with receiving naloxone in the previous six months [118].

In conclusion, the usage of opioids as related to suicidality is clearly a very complicated and multifactorial relationship. Multiple studies studying the risk of suicide as related to opioid use are presented in Table 2. The general trend of decreasing opioid prescriptions, as well as the increased availability of resources for those with an opioid use disorder, all signal a general trend of better addressing this risk. More research in the field of mental health screening for the prescribers of opioids as well as early interventions for potential opioid use disorder patients are likely needed moving forward.

### 4.3. Gabapentinoids

The antiepileptics gabapentin and pregabalin are routinely used for the treatment of chronic neuropathic pain, and particularly diabetic neuropathy. While systematic reviews have validated their efficacy [123,124], experts are increasingly concerned about how ubiquitous their use has become [125]. By 2016, 64 million prescriptions were issued for gabapentin, making it the 10th most prescribed drug in the US market. This constitutes a rise of over 160% compared to 2012. Pregabalin sales generated USD 4.4 billion in 2106, more than doubling the sales value since 2012 [125]. This phenomenon was simultaneously occurring, with different magnitudes, across the world. During the period of 2014–2019 in France, gabapentin and pregabalin prescriptions increased by 17.5% and 16.6%, respectively [126]. In Scotland, gabapentin prescriptions quadrupled between 2000 and 2016 [127].

While the prevalence of conditions treated with these medications continues to increase, the opioid epidemic was equally, if not primarily, responsible for the increase in physicians prescribing gabapentinoids. Although the most common side effects of antiepileptics are well documented, dose-dependent, and reversible, the implication of gabapentinoids in drug misuse, self-harm, and suicidality has come into focus in recent years [8,125,126].

The association between antiepileptics and suicidality was described by multiple systematic reviews, prompting the FDA to issue a warning for 11 drugs, including gabapentin, in 2008. However, despite a growing body of literature on the topic, gabapentinoids are not considered controlled substances in Europe, Canada, Australia, or the United States at the federal level, although some states have begun to reclassify them individually [128]. The UK is a noteworthy exception, having reclassified gabapentin and pregabalin as controlled drugs in 2019 [129].

Over the past several years, multiple studies have been published analyzing the association between gabapentinoids use and suicidality, self-harm, misuse, and overdose at the population level. The results of these studies are summarized in Table 3.

Studying the association between this class of medication and the outcomes amongst chronic pain patients is complicated by two main factors. The first is the overlap between metal health and psychiatric disorders and chronic pain [137]. The second is the polypharmacy nature of these patients, which makes attributing specific outcomes or safety concerns to individual drugs challenging. However, in late 2019, the FDA issued a warning in light of the mounting evidence that gabapentinoids may cause respiratory suppression and are most dangerous when concomitantly used with other respiratory depressants, such as opioids [138,139].

### 4.4. Benzodiazepines

As previously mentioned, chronic pain patients are often polypharmacy patients, managing their symptoms with several treatment modalities. Especially in the United States, opioids take the main place in the treatment options for chronic pain [140]. However, the co-administration of benzodiazepines with opioids has been a prominent pain relief strategy for these patients [141,142,143], leading to concerns surrounding interaction and adverse-event amplification when used concomitantly [144,145,146,147,148,149]. A study found that patients with a co-prescription of opioids and benzodiazepines were more likely to be diagnosed with depression, PTSD, and bipolar disorder and were more likely to be prescribed additional medication, such as antidepressants [150].

A recent epidemiological study looking at the concomitant use of opioids and benzodiazepines showed an increased risk of suicide attempts, intentional self-harm, and overdose, but interestingly, all of these were attributed largely to the effects of benzodiazepines [151]. However, not all interaction between these groups of medications can be excluded. A recent study reported a higher incidence of all-cause mortality and overdose mortality in patients using both groups of medications compared to those that used only benzodiazepines [152], a relationship further established in other publications [153].

The most common conditions that are primarily treated with benzodiazepines that are linked to possible suicidality are anxiety and insomnia [154,155]. Treating these conditions would lead to a logical decrease in suicidality, but what happens when the treatment option itself is a possible risk factor for suicide?

Various instances in the literature suggest a very strong association between the use of benzodiazepines and suicidality and suicide attempts. However, many of these studies fall under possible indication bias, linking the condition being treated to possible suicidality rather than the pharmacological treatment option itself [156,157,158,159,160]. Minimizing such biases, by using cohorts [161,162,163] and case–control studies [164], yielded similar positive correlations.

Benzodiazepines have been shown to increase aggression and impulsivity [165], both of which appear to be correlated to suicidal tendencies [166]. Recent studies showed a dose-dependent relationship with the incidence of suicidality, both in short- and long-term benzodiazepine use [158,162,167,168]. A 2015 case-cohort study showed a clear correlation between a receipt of concurrent benzodiazepine and the risk of death from overdose, following a dose–response fashion [146].

Benzodiazepine safety and misuse practice have been of great national interest worldwide, highly due to their continuous prevalent use. A highlight of recent studies and their key points concerning these topics can be found in Table 4.

### 4.5. Other Medication Groups

In this paper, we addressed some of the most common medication classes that are indicated in the treatment of chronic pain. In this section, we briefly review some miscellaneous medication classes that are implicated in the relationship between suicide and the pharmacological treatment of pain. A 2019 publication used commercial claims data to determine the odds ratio of an increase in suicidal events following the initiation of a medication, excluding overdoses. The paper studied all drugs with over 3000 filled prescriptions per year, yielding 922 medications [175]. Out of the 10 prescriptions that were most associated with post-exposure increases in suicidal events, 7 are indicated for managing pain. Of these seven medications, three belong to classes of medications that we did not yet discuss in this paper: the barbiturate combination pill of acetaminophen/butalbital/caffeine, the 5-HT2 antagonist cyclobenzaprine, and the glucocorticoid prednisone.

#### 4.5.1. Barbiturates

The combination acetaminophen/butalbital/caffeine pill uses the analgesic effects of acetaminophen with the barbiturate butalbital and caffeine for the treatment of tension headaches. A tension headache is a type of headache that causes pain in a band around the head bilaterally [176]. Out of all the medications studied in a 2019 paper, the combination acetaminophen/butalbital/caffeine pill was associated with the highest odds ratio of suicidal event increase after exposure, 1.68 (95 CI 1.16–2.44), with the paper able to study 1,154,666 patients that were prescribed this combination [175]. Headaches are one of the most common forms of pain, both acute and chronic. About 15.8% of the global population experiences headaches each day [177]. More specifically, a population study conducted in Denmark found a prevalence of chronic tension headaches at 2–3% [178]. A study of veterans with chronic pain found that, among the patients studied, those with chronic headaches had the highest incidence of suicide attempts, ranging from 329 to 491 per 100,000 [179].

The combination acetaminophen/butalbital/caffeine pill has been shown to be effective at anxiolysis in the tension headache population [180]. Although the pathogenesis of tension headaches has not been fully elucidated, there is likely a relationship with mental health diagnosis, with one study showing an 84% overlap in patients with tension headaches and psychiatric diagnosis [181]. This masking of psychological symptoms along with analgesia creates a risk of over usage in times of stress, in turn raising the risk of both overdose as well as a possible withdrawal-associated psychiatric crisis [182]. Barbiturate prescriptions have been on the decline for decades, but prescribers should be vigilant in monitoring patients for comorbidities as well as medication misuse.

#### 4.5.2. Cyclobenzaprine

Cyclobenzaprine is a skeletal muscle relaxant marketed for the use of musculoskeletal pain and/or spasms, although it is also used for the treatment of fibromyalgia off-label [183]. A 2019 study that found that the association between cyclobenzaprine and suicide had an odds ratio of 1.34 (95% CI 1.06–1.68) with 7,487,505 patients. Of note, the study found a smaller risk of suicidality in men compared to women, with an odds ratio of 1.21 (0.96–1.58) vs. 1.39 (1.13–1.71), respectively [175].

In a study of the most frequently seen causes of single- or multiple-product suicidal poisoning, cyclobenzaprine was found to be the 19th most common, single-product suicidal poison and the 15th most common in polypharmacy poisonings [184]. Unfortunately, there is an overall paucity of research investigating the relationship between cyclobenzaprine usage and suicidality, and more research is needed to help to better understand how to best guide prescribing practices and patient monitoring.

#### 4.5.3. Prednisone

Prednisone is a glucocorticoid medication used for a wide variety of pathologies. By decreasing inflammatory responses in the body, steroid medications can help with many painful rheumatic conditions. Prednisone was found to carry an overall odds ratio of suicidality of 1.33 (1.10–1.61), with higher rates in men 1.42 (1.07–1.88) vs. women 1.33 (1.08–1.64). The paper was able to study 10,667,620 patients [175]. Two commonly cited papers assessing the incident rate of psychiatric events in patients treated with steroids described a rate of severe psychiatric adverse events at 6% in patients treated at high doses, while another cohort study found an incidence of mania or depression as high as 36% among patients treated with high-dose steroid regimens [185,186]. In a trial of children being treated with steroids while hospitalized for leukemia, researchers found a behavioral adverse event incidence of 6% [187].

While there is still more research to be conducted regarding the exact incidence of psychiatric side effects in patients being treated with steroids, another important question left unanswered is the mechanism by which these effects arise. One theory states that exogenous glucocorticoids may influence the brain’s synthesis of GABAergic steroids, causing a cascade of side effects. By altering the synthesis of GABAergic steroids, the hypothalamic–pituitary–adrenal axis is liable to fall out of regulation, leading to systemic effects [188]. This theory is potentially bolstered by a case series of steroid-induced psychiatric crisis being stabilized by sodium valproate [189], a drug whose mechanism of action is thought to increase the levels of GABA [190]. In this case series, 20 patients all receiving steroids developed psychiatric symptoms that were rapidly abated after starting the valproate therapy. Moving forward, more research is needed to best understand how to monitor patients to prevent adverse event outcomes, as well as how to best screen patients who have a higher risk of crisis during treatment.

## 5. Conclusions

In conclusion, in this paper, we addressed the relationship between the different classes of medications that are often prescribed to the chronic pain population and how their usage impacts the psychological safety of patients. We also discussed the epidemiological, socio-economic, and pharmacodynamic interactions between variables such as psychiatric diagnoses, drug–drug interactions, race, and sex. Furthermore, this study identified current epidemiological phenomena and trends pertaining to drug safety and the risk of suicidality in chronic pain patients. Although it would be impossible to address every variable that contributes to suicidality in pain patients, the topics mentioned in this review should provide prescribers a heightened awareness to not just monitor a patient’s psychical health, but their mental health as well. Perhaps the most significant finding is that there is a growing number of patients that utilize these medications, which, combined with a shift away from opioids due to the epidemic, has uncovered novel safety concerns. This not only highlights the importance of pharmacovigilance, and further research, but also underscores the importance of pharmaco-surveillance programs, which are, unfortunately, lacking.

## Figures and Tables

**Table 1:** Findings from national databases concerning suicide rates and suicidality in association with antidepressant use.

Year	Publication	Location	Data Set	Key Findings
2022	Yang et al. [94]	Sweden	Swedish National Register, women born in 1960–1995; Prescribed Drug Register of 1987–2011	Simultaneous use of antidepressants and hormonal contraceptives showed a decrease in suicidality compared to women without treatment.
2021	Amendola et al. [64]	Switzerland, Italy, Austria	WHO Mortality Database, 1951–2013/15/16	No changes in suicide rates since the introduction of antidepressants (1960); Steep rise in prescription since the introduction of SSRIs (1990).
2015	Christiansen et al. [93]	Denmark	Variety (Danish Register of Causes of Death; National Patient Register; Danish Psychiatric Central Register), 1977–2011	Redeeming SSRIs was associated with a rise in suicide attempts; This correlation was most evident in the first 3 months of withdrawing the medication.
2013	Björkenstam et al. [92]	Sweden	Swedish Causes of Death Register, 2007–2010	Incidence of suicide attempts rose after the initiation of the SSRI treatment; This correlation was most evident at the beginning stages of the treatment.
2013	Makris et al. [95]	Sweden	Swedish Cause of Death Register, 1992–2003	Higher seasonal variation in suicide amongst men on SSRIs; The style of suicide was more violent in this group.
2005	Ludwig et al. [96]	Variety (27 countries)	World Health Organization (WHO) Mortality Database, 1999–2005	Increase in SSRI sales was followed by a decrease in suicide rates.

**Table 2:** Findings from national databases concerning suicide rates and suicidality in association with opioid use.

Year	Publication	Location	Data Set	Key Findings
2023	Huang et al. [119]	UK	UK Biobank samples	There are strong genotypic and phenotypic relationships between opioid use disorder and suicide attempts, even when controlling for patients with a prior psychiatric disease status.
2022	Agyemang et al. [120]	New Mexico, USA	New Mexico Youth Resiliency and Risk Survey	The rate of opioid use was 12% and the rate of suicide attempt was 14% among New Mexico high school students from the American Indian and Alaskan native population; High social support levels among opioid users was protective against suicide attempt.
2019	Samples et al. [121]	USA	2015–2016 National Survey on Drug Use and Health	Opioid use without misuse (in patients with prescriptions) was associated with lower odds of suicidal ideation compared to patients with opioid misuse (odds ratio of 0.57).
2017	Hollingsworth et al. [103]	USA	CDC Multiple Cause of Death files, 1999–2014	A 1% increase in unemployment per county raises the opioid death rate by 3.6% and opioid-related emergency department visits by 7%.
2016	Ilgen et al. [122]	USA	Veterans Affairs health care system treatment records and the National Death Index	Higher opioid doses were associated with an elevated suicide risk; Hazard ratio of suicide was 1.48, in the range of 20–50 mg morphine equivalents per day; Hazard ratio of suicide was 1.69, in the range of 50 and 100 mg morphine equivalents per day.

**Table 3:** Findings from national databases and population studies concerning misuse, self-harm, overdoes, and suicidality in association with gabapentinoids.

Year	Publication	Location	Data Set	Key Findings
2023	Antunovic et al. [130]	Serbia	National Poison Control Center of Serbia, 2012–2022	357 cases of gabapentinoid poisoning; 30.2% of cases attributed to misuse; 40.9% classified as suicide-related events.
2023	Koseki et al. [131]	Japan	Japanese Adverse Drug Event Report, 2004–2021	Reported odds ratio of gabapentin use in suicide-related events was 3.86, 95% CI 1.82–8.16.
2020	Torrance et al. [127]	Scotland	National Records of Scotland and Tayside Drug Death Databases, 2007–2016	Age-standardized death rate in patients who were prescribed gabapentinoids was double than that of the general population (relative risk 2.16, 95% CI 2.08–2.25); Both gabapentin and pregabalin were increasingly contributing towards drug related deaths at the national level.
2020	Reynolds et al. [132]	USA	National Poison Data System, 2013–2017	Reported gabapentin exposures increased by 72.3% and isolated exposures increased by 67.1%; Isolated abuse/misuse increased by 119.9%; Intentional suspected suicide attempts with isolated gabapentin exposures increased by 80.5%.
2019	Evoy et al. [133]	USA	Food and Drug Administration Adverse Event Reporting System (FAERS), 2012–2016	Of all the adverse drug events reported regarding gabapentin and pregabalin, 5.7% and 10.2% were attributed to abuse, respectively.
2018	Daly et al. [134]	Ireland	National Self-Harm Registry Ireland, 2007–2015	The percentage on intentional drug overdoses involving gabapentinoids increased from 0.5% to 5.5% over the analyzed period; Admission to hospital after intentional drug overdoses, including gabapentinoids, was significantly more common (49.4% vs. 41.4%, *p* ≤ 0.001).
2010	Patorno et al. [135]	USA	HealthCore Integrated Research Database (HIRD), 2001–2006	The risk of attempted or completed suicide was meaningfully increased for patients who were prescribed gabapentin (hazard ratio (HR), 1.42; 95% CI, 1.11–1.80).
2010	Gibbons et al. [136]	USA	PharMetrics Medical Claims Database, 2000–2006	Gabapentin was found not to increase the risk of suicide attempts in non-psychiatric populations and was associated with a reduction in suicide attempt risk in patients with psychiatric disorders.

**Table 4:** Findings from national databases and population studies concerning misuse, overdoes, and suicidality in association with benzodiazepines.

Year	Publication	Location	Data Set	Key Findings
2023	Tournier et al. [169]	France	French Reimbursment Healthcare System (SNDS), 2013–2016	The recent use of benzodiazepines has been linked to the occurrence of both suicide and suicide attempts requiring hospitalization.
2021	Kyu Oh et al. [170]	South Korea	South Korean National Health Insurance Service, 2010–2015	Benzodiazepine use was associated with an increased 5-year all-cause mortality risk.
2019	Maust et al. [171]	United States	National Survey of Drug Use and Health, 2015–2016	Young adults misuse benzodiazepines without prescription; older adults use their prescriptions more often; Amongst all groups, misuse to help to relax and sleep were the most common reasons; Benzodiazepine misuse was strongly associated with opioid and stimulant misuse.
2018	Cadogan et al. [172]	Ireland	Irish General Medical Services (GMS), 2005–2015	Benzodiazepine prescription decreased with the introduction of Z-drugs; Both benzodiazepines and Z-drugs still follow the long-term 4-week prescription practices.
2018	Potočnjak et al. [173]	Croatia and Sweden	Agency of Medical Products and Medical Devices; Swedish eHealth Agency, 2014–2015	The misuse and abuse of benzodiazepines has been shown in socio-economically weaker standing countries (Croatia in comparison to Sweden); The price and availability of drugs highly influence the practices of prescription within a country.
2016	Murphy et al. [174]	Canada	International Narcotics Control Board (INCB); The Canadian Alcohol and Drug Use Monitoring Survey (CADUMS), 1995–2015	The rates of benzodiazepine misuse and related mortality have a steady rate without a significant decline; There are significant elder-associated adverse effects and events, such as accidents, injuries, cognitive decline, and sleep disturbances.

## Data Availability

Data sharing is not applicable.
